# UCtracker: A Deep Learning–Based DNA Methylation Model for Noninvasive Diagnosis and Recurrence Surveillance of Urothelial Carcinoma in a Prospective Study

**DOI:** 10.1002/advs.76710

**Published:** 2026-08-03

**Authors:** Shengwei Xiong, Gaojie Li, Yucai Wu, Yuan Liang, Heng Guo, Yu Zhang, Gengyan Xiong, Long Tian, Xin Zhang, Ye Tian, Zhengguo Ji, Bin Guo, Yue Shi, Jian Fan, Zhihua Li, Yanqing Gong, Shiming He, Xuesong Li, Weimin Ci, Liqun Zhou

**Affiliations:** ^1^ Department of Urology Institute of Urology Beijing Key Laboratory of Precision Medicine and Innovative Translation for Urogenital Diseases Peking University First Hospital National Urological Cancer Center Peking University Beijing China; ^2^ China National Center for Bioinformation Beijing Institute of Genomics Chinese Academy of Sciences Beijing China; ^3^ Institute for Regenerative Biology and Medicine Chinese Institutes for Medical Research Beijing China; ^4^ Department of Urology Beijing Chao‐Yang Hospital Institute of Urology Capital Medical University Beijing China; ^5^ Department of Urology Beijing Friendship Hospital Capital Medical University Beijing China; ^6^ Department of Urology Chinese PLA General Hospital Beijing China; ^7^ Department of Urology First Affiliated Hospital of Henan University Kaifeng China

**Keywords:** deep learning, DNA methylation, noninvasive detection, surveillance, urothelial carcinoma

## Abstract

Noninvasive diagnosis and longitudinal surveillance of urothelial carcinoma (UC) remain clinically challenging. Here, we developed and prospectively validate UCtracker, a urine DNA methylation–based deep learning model for UC detection and postoperative recurrence monitoring. UC‐specific differentially methylated regions (DMRs) were identified by high‐depth whole‐genome bisulfite sequencing of UC tissues, paired adjacent normal tissues, and non‐tumor urine samples. UCtracker was constructed using the top 2000 hypomethylated DMRs and a convolutional neural network–bidirectional long short‐term memory architecture. In an internal validation cohort (*n* = 165), UCtracker achieved a sensitivity of 94.6% and a specificity of 94.4%, and maintained high performance in an independent multicenter cohort (*n* = 55), with a sensitivity of 90.6% and a specificity of 91.3%. UCtracker showed higher sensitivity than UroVysion fluorescence in situ hybridization for T1 tumors, high‐grade tumors, and bladder UC. Subsampling analyses demonstrated stable diagnostic performance even at ultralow sequencing depths. In postoperative surveillance, longitudinal urine profiling of 131 samples from 48 UC patients detected 94.1% of recurrence events and identified recurrence up to 250 days before clinical confirmation. These findings support UCtracker as a highly accurate and cost‐effective urine‐based tool for UC diagnosis, postoperative surveillance, and personalized patient management.

## Graphical abstract

We developed UCtracker, a urine DNA methylation–based deep learning model, for noninvasive diagnosis and postoperative surveillance of urothelial carcinoma. UCtracker demonstrates high diagnostic accuracy, robustness at ultralow sequencing depth, early recurrence detection, and dynamic risk‐stratified monitoring of molecular residual disease, supporting its potential for cost‐effective and personalized management of patients with urothelial carcinoma.

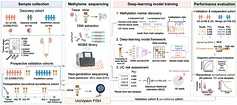

## 1 Introduction

Urothelial carcinoma (UC), the ninth most common malignancy worldwide, arises primarily from the bladder (90%–95%) or upper urinary tract (5%–10%) [1]. UC is characterized by a high propensity for recurrence and progression, imposing a substantial clinical and economic burden worldwide. Approximately 75% of UC of the bladder (UCB) presents as non‐muscle invasive at diagnosis; however, recurrence rates remain high after transurethral resection of bladder tumor, ranging from 15%–61% at 1 year and 31%–78% at 5 years [2]. In addition, approximately two‐thirds of patients with upper tract urothelial carcinoma (UTUC) have muscle‐invasive disease at diagnosis, and about 9% present with metastatic disease [3]. Despite radical surgery, intravesical recurrence occurs in approximately 29% of UTUC cases [4]. Early detection of UC and effective surveillance for recurrence or progression are therefore critical, yet current clinical practice relies heavily on ureteroscopy or repeated cystoscopies. This dependence on invasive procedures has driven substantial interest in the development of accurate, noninvasive biomarkers for UC detection and longitudinal monitoring.

Urine represents an optimal liquid biopsy for UC, as direct tumor–urine contact enables continuous shedding of tumor cells and tumor‐derived DNA. Aberrant DNA methylation emerged as a hallmark of urothelial carcinogenesis and is highly stable and detectable in urine‐derived DNA, making it a leading biomarker for noninvasive UC detection [5]. A recent meta‐analysis identified several promising urinary DNA methylation markers for UC detection, including *SALL3*, *PENK*, *ZNF154*, *VIM*, and *POU4F2*, with moderate diagnostic accuracy [6]. Clinically, Bladder EpiCheck, a 15‐CpG methylation assay, has been approved for surveillance of non–muscle‐invasive UCB and demonstrates acceptable diagnostic accuracy for UTUC [7, 8, 9]. However, the marked genomic and epigenomic heterogeneity of UC limits the robustness and generalizability of single‐gene or small‐panel assays. Genome‐wide DNA methylation profiling enables the extraction of complex, disease‐specific epigenetic signatures and improves tumor detection performance [10, 11]. In this context, we previously developed GUseek, an integrative machine learning framework that jointly analyzes DNA methylomes and copy‐number variations (CNVs) derived from urinary sediment using whole‐genome bisulfite sequencing (WGBS) data, achieving high accuracy in detecting genitourinary cancers [12]. We subsequently improved this approach into UCseek, which demonstrates high accuracy for UC diagnosis and longitudinal disease monitoring in retrospective cohorts [13].

Urinary tumor DNA (utDNA) from urinary sediment and cell‐free DNA (cfDNA) from urinary supernatant provide overlapping yet complementary genomic information, showing high concordance in somatic mutations and CNVs, and their integration may enhance tumor detectability [14, 15]. In addition, DISMIR, a deep learning framework that integrates DNA sequence and single‐read methylation patterns from WGBS data, has demonstrated the capability of ultrasensitive and robust cancer detection [16]. In this study, we developed a novel approach named UCtracker, a DISMIR‐based model constructed using whole urine–derived DNA (cfDNA and utDNA). We further validated the performance of UCtracker for UC diagnosis and recurrence surveillance in prospective cohorts.

## 2 Methods

### 2.1 Study Design and Participants

We conducted a prospective, blinded, multicenter cohort study. The study design and analytical workflow were depicted in Figure 1A,B. The discovery set for methylation marker identification comprised surgically resected tumor and adjacent non‐tumor tissues from participants with UC, as well as urine samples from individuals with non‐tumor urological diseases. All samples in the discovery set were collected at Peking University First Hospital (PKUFH). Subsequently, prospective validation cohorts were assembled to evaluate the performance of the developed model, incorporating participants recruited from multiple centers, including PKUFH, Beijing Friendship Hospital (BJFH), and Beijing Chao‐Yang Hospital (BJCH). Investigators were blinded to all clinical information and reference standard results. Histopathological examination of surgical tissue specimens served as the reference standard for diagnostic classification. Detailed inclusion and exclusion criteria of the participants are provided in the Supplementary Methods.

**FIGURE 1:**
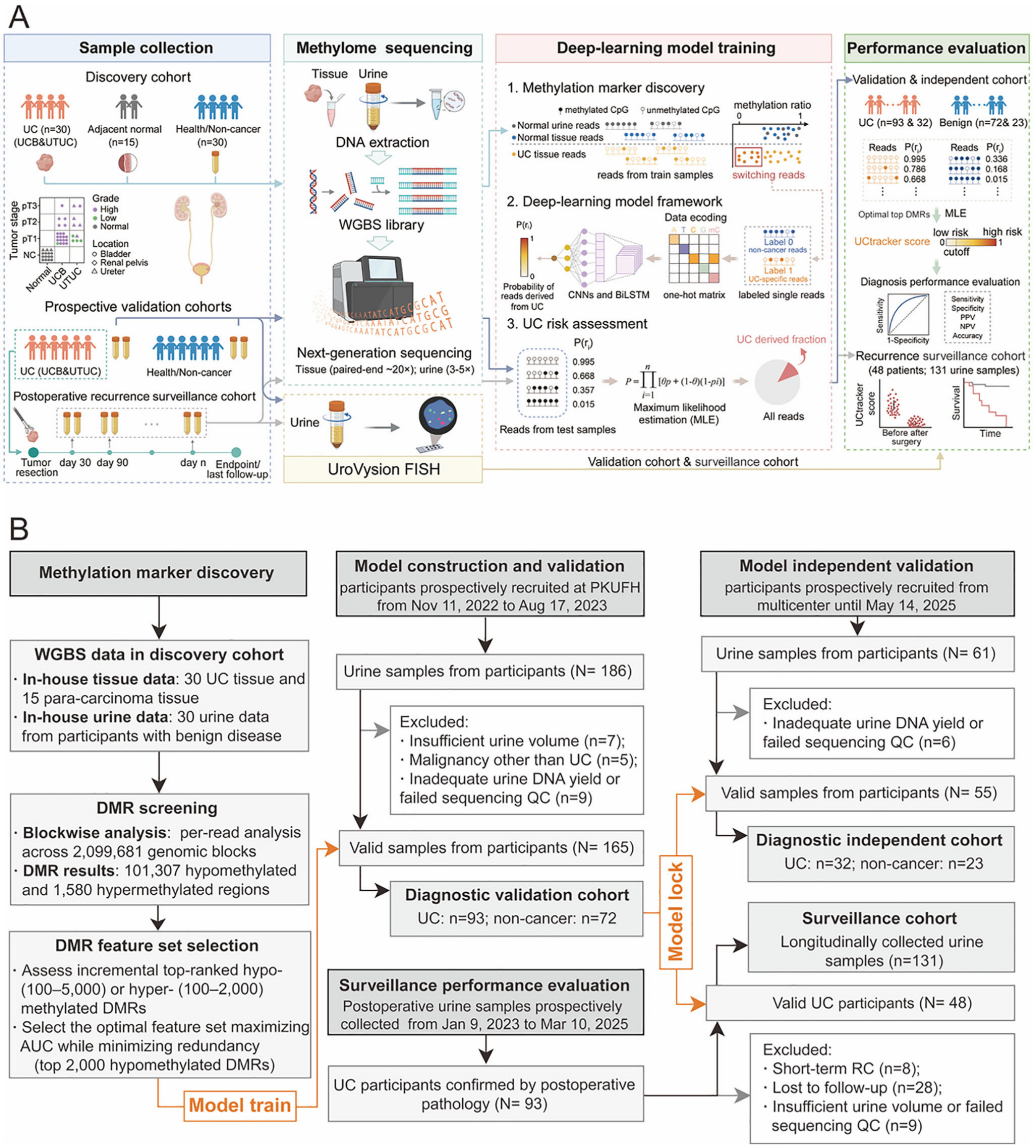
Overview of study design and workflow. (A) Schematic of the study design. (B) Flow diagram of participant enrollment and DMR feature set selection. Abbreviation: UC, urothelial carcinoma; UCB, urothelial carcinoma of the bladder; UTUC, upper tract urothelial carcinoma; CNNs, convolutional neural networks; BiLSTM, bidirectional long short‐term memory; WGBS, whole‐genome bisulfite sequencing; utDNA, urinary tumor DNA; cfDNA, cell‐free DNA; FISH, fluorescence in situ hybridization; DMR, differentially methylated region; QC, quality control; RC, radical cystectomy; AUC, the area under the receiver operating characteristic curve.

To evaluate the model's recurrence surveillance performance, we constructed a longitudinal cohort consisting of eligible participants with pathologically confirmed UC after surgery. Participants who underwent short‐term radical cystectomy, were lost to follow‐up, provided samples with insufficient volume or failing sequencing quality control, were excluded from the final analysis. Histopathological findings from surveillance cystoscopic biopsy specimens or from surgical specimens obtained during subsequent operations for recurrent disease were used as the reference standard for recurrence monitoring. The study was approved by the ethics committees of the three participating centers (Approval No. 2022‐209‐002, 2024‐P2‐060‐01, and 2024–93 for PKUFH, BJFH, and BJCH, respectively), and written informed consent was obtained from all participants. The study was registered at chictr.org.cn (ID: ChiCTR2200063932) before participant enrollment.

### 2.2 Sample Collection and Processing

To establish a high‐confidence UC‐derived methylation reference for model training, we collected surgically resected tumor tissues and matched adjacent para‐carcinoma tissues from both UCB and UTUC. For UCB, specimens were obtained during transurethral resection of bladder tumor or radical cystectomy. For UTUC, tissues were collected during radical nephroureterectomy. All specimens were confirmed as UC by postoperative pathological examination. Fresh tissues were snap‐frozen and stored at −80°C before DNA extraction.

Voided urine was prospectively collected on the day of surgery and during postoperative surveillance. Each urine sample was assigned a unique identifier to ensure blinded assessment. Of the total 150 mL urine volume, 50 mL was allocated for urine DNA extraction and 100 mL for UroVysion fluorescence in situ hybridization (Figure 1A). For postoperative surveillance of UC patients, urine samples were collected at the 1–3‐month postoperative landmark and subsequently at guideline‐recommended follow‐up intervals for non‐muscle invasive UCB and UTUC until intravesical recurrence [3, 17]. Urine conditioning buffer was added immediately after collection to stabilize cellular and cfDNA. Samples were stored at −80°C and subsequently processed in batches for DNA extraction.

### 2.3 DNA Extraction and Library Construction

Genomic DNA from tissue specimens was extracted using the Quick‐DNA Plus Kit (Zymo Research, D4705), and whole urine DNA (both cellular and cell‐free) was isolated using the Quick‐DNA Urine Kit (Zymo Research, D3061), following the respective manufacturer's protocols. DNA samples were quantified using Qubit 4.0 using a dsDNA HS Assay Kit (Thermo Fisher Scientific, Q32854). All extracted DNA was stored at −20°C until further processing. Input DNA (20–100 ng) was fragmented to an average size of 300–400 bp using a Covaris S220 focused ultrasonicator (Covaris, Woburn, MA, USA) following the manufacturer's optimized settings. Fragment size distribution was confirmed using a Bioanalyzer (Agilent Technologies, Santa Clara, CA, USA). Fragmented DNA was then subjected to bisulfite conversion using the EpiArt DNA Methylation Bisulfite Kit V2 (Vazyme, EM102) according to the manufacturer's protocol. Methylation libraries were subsequently generated with the EpiArt DNA Methylation Library Kit for Illumina V3 (Vazyme, EM102), which utilizes a single‐strand ligation–based workflow optimized for low‐input material (100 pg–2 ng). In brief, bisulfite‐converted DNA was denatured and sequentially ligated to 3′ and 5′ adapters, followed by primer extension and limited‐cycle PCR to produce Illumina‐compatible libraries.

### 2.4 Methylation Sequencing and Data Processing

Sequencing libraries were sequenced on the Illumina NextSeq 6000 platform to generate 150 bp paired‐end reads, with an average sequencing depth of approximately 20× for tissue DNA and 3×–5× for urine DNA. Raw sequencing reads underwent quality control using fastp (version 1.0.1) [18] and cutadapt (version 5.1) [19]. To eliminate potential adapter sequences and low‐quality bases, the first 10 nucleotides from the 5′ end of Read 2 and the last 10 nucleotides from the 3′ end of Read 1 were trimmed prior to downstream alignment. Clean reads were subsequently aligned to the human reference genome (hg19) using BS‐Seeker2 (version 2.1.8) [20] with Bowtie2 (version 2.3.5.1) [21] under default parameters. Aligned reads were sorted, and PCR duplicates were removed using SAMtools (version 1.18) [22] and sambamba (version 1.0.1) [23]. Sequencing depth and genome coverage were assessed using mosdepth (version 0.3.10) [24], and bisulfite conversion efficiency was evaluated based on alignment data using pysam (version 0.15.3). For each aligned read, CpG sites were identified and encoded in genomic order as a binary methylation pattern (1 = methylated, 0 = unmethylated).

### 2.5 DMRs Calling Based On Read‐Level Methylation

Given the high epigenetic similarity between UTUC and UCB demonstrated by previous whole‐methylome comparisons [12, 25], UC tissue samples from both anatomical sites were pooled to identify UC‐specific DMRs in this study (Figure 1A and Figure S1A). DMR screening was performed based on per‐read methylation measurements across 2 099 681 genomic blocks [26]. These blocks correspond to methylation linkage domains previously defined from 39 sorted cell types spanning 205 healthy tissue samples, representing regions of coordinated methylation variation in normal tissues [26]. For each block, the methylation level and its distribution across reads were calculated and used for subsequent DMR detection [27]. UC‐specific DMRs were identified by comparing UC tissue samples with controls (non‐tumor urine and adjacent normal tissues). Genomic blocks showing an abnormal methylation‐read ratio > 0.25 and a false discovery rate (FDR)‐adjusted *p* < 0.05 in tumor samples relative to controls were defined as DMRs.

### 2.6 Deep Learning Model Training

A deep learning framework integrating convolutional neural networks (CNNs) and bidirectional long short‐term memory (BiLSTM) layers was constructed to classify sequencing reads according to their tissue of origin [16, 27]. Reads were labeled as UC‐derived (“1”) or non‐tumor‐derived (“0”) and encoded into one‐hot matrices that represented both nucleotide identity and methylation status (Figure 1A and Figure S1B). The model architecture included one‐dimensional convolutional, max pooling, dropout, BiLSTM, and fully connected layers. Convolutional modules captured local sequence–methylation features, while BiLSTM layers extracted long‐range dependencies. A sigmoid activation function produced a probability score between 0 and 1, reflecting the likelihood of each read being UC‐derived (Figure 1A and Figure S1C).

### 2.7 UC‐Derived Reads Fraction Estimation

To estimate the proportion of UC‐derived reads within each sample, a maximum‐likelihood estimation (MLE) approach was applied based on the probability scores generated by the trained deep learning model (Figure 1A and Figure S1D). For each read *i*, the model produced a probability *p_i_* representing the likelihood that read *i* was UC‐derived. Reads were assumed to be independent observations arising from a two‐component mixture of UC‐derived and non‐tumor‐derived reads. Let *θ* denote the proportion of UC‐derived reads in the sample. The likelihood of observing read *i* is:

$$L_{i}\;\left(\theta \right)=\theta p_{i}+\left(1-\theta \right)\left(1-p_{i}\right)$$

Here, *p_i_* is the model's estimate that read *i* is UC‐derived, and (1 − *p_i_*) is the corresponding probability that it is non‐tumor‐derived. For a sample containing *n* reads within UC‐specific DMRs, the overall likelihood function is the product of the per‐read likelihoods:

$$L\left(\theta \right)=\prod_{i=1}^{n}L_{i}\left(\theta \right)=\prod_{i=1}^{n}\left[\theta p_{i}+\left(1-\theta \right)\left(1-p_{i}\right)\right]$$

The value of *θ* was estimated by maximizing L(θ) over the interval [0, 1] using a grid search with a resolution of 0.001. A higher estimated *θ* indicates a greater fraction of UC‐derived reads in the sample and serves as a proxy for malignancy burden.

### 2.8 Feature Set Selection and Hyperparameter Tuning

To determine the optimal feature set for the deep learning classifier, we performed hyperparameter optimization on the validation cohort, specifically for feature count selection. Candidate hypomethylated and hypermethylated DMRs were independently evaluated. For each DMR category, regions were ranked by the magnitude of differential methylation (Figure 1B). Using the validation cohort, we systematically assessed classifier performance with increasing numbers of top‐ranked DMRs, according to the total number of DMRs available in each category. For each feature set, the same neural network architecture was trained, and the area under the receiver operating characteristic curve (AUC) was calculated on the validation cohort. The optimal number of features was selected based on the best balance of AUC and feature redundancy (Figure 1B). The independent validation cohort was further used for final unbiased performance evaluation.

### 2.9 Optimal Cutoff Determination

Following construction of the deep learning model, the optimal cutoff value was determined by maximizing Youden's *J* statistic [28]. Youden's index summarizes the overall discriminative performance of a diagnostic test by assigning equal weight to sensitivity (*Se*) and specificity (*Sp*). The index was calculated across all possible cutoff thresholds *c* as follows:

$$J\;\left(c\right)=Se\left(c\right)+Sp\left(c\right)-1$$

This index can be equivalently expressed in terms of true positives (*TP*), false positives (*FP*), true negatives (*TN*), and false negatives (*FN*) as follows:

$$J\;\left(c\right)=\frac{TP}{TP+FN}+\frac{TN}{TN+FP}-1$$

The value of Youden's index ranges from −1 to 1, with higher values indicating superior discriminative performance. In the receiver operating characteristic (ROC) analysis, the cutoff c* that maximizes *J*(*c*) corresponds to the operating point on the ROC curve closest to the top‐left corner. This optimal cutoff c* was therefore selected as the final decision threshold for tumor detection (Figure S1E).

### 2.10 Fluorescence In situ Hybridization in Urine Samples

Fluorescence in situ hybridization (FISH) was performed on exfoliated urothelial cells from urine samples using the UroVysion Kit (GP Medical Technologies Ltd., Beijing, China), an FDA‐approved test for the detection and surveillance of bladder cancer in patients with hematuria, according to the manufacturer's protocol. Briefly, exfoliated cells were harvested by centrifugation, subjected to hypotonic treatment, and fixed in Carnoy's solution (methanol: acetic acid, 3:1), then dropped onto slides and air‐dried. Slides were pretreated with 2× caline–sodium citrate buffer, digested with pepsin, post‐fixed in 1% formaldehyde, and hybridized with probes targeting chromosomes 3 (CEP3, green), 7 (CEP7, red), 17 (CEP17, aqua), and 9p21 (p16, gold). Stringent washes were performed, and nuclei were counterstained with DAPI. At least 100 morphologically intact cells were scored per case. Abnormal results were defined as gains of chromosomes 3, 7, or 17 (≥3 signals in ≥10% of analyzed cells) or homozygous 9p21 deletion (0 signals in ≥20% of analyzed cells), and samples exhibiting abnormalities in at least two probe targets were classified as positive for UC [29, 30, 31]. All slides were independently reviewed by two board‐certified pathologists blinded to clinical data, with discrepant cases resolved by consensus.

### 2.11 Statistical Analysis and Metrics Evaluation

Model performance was evaluated using sensitivity, specificity, accuracy, positive predictive value (PPV), negative predictive value (NPV), and corresponding 95% confidence intervals (CIs). The overall discriminative ability was quantified by the AUC. To evaluate the influence of sequencing depth on model performance, mapped reads were systematically down‐sampled to 1%, 3%, 5%, 7%, 9%, 10%, 30%, 50%, 70%, and 90% of the original sequencing volume. Model performance at each depth was evaluated by sensitivity, accuracy, and the correlation between model scores derived from down‐sampled and original sequencing depth data. For survival analysis, recurrence‐free survival (RFS) was compared between groups using the Kaplan–Meier method and the log‐rank test. The association between the model score and postoperative recurrence was first evaluated by univariable Cox proportional hazards regression.

Differences in model scores across clinical subgroups were analyzed using the Wilcoxon rank‐sum test or Kruskal–Wallis test, as appropriate. The two‐sided *p* < 0.05 was considered statistically significant. All statistical analysis were performed using R (version 4.4.2) and Python (version 3.10.5).

## 3 Results

### 3.1 Participants Characteristics

This study was conducted in three main stages: (1) methylation marker discovery, (2) deep learning model development and diagnostic performance evaluation, and (3) recurrence surveillance performance evaluation (Figure 1A,B). To identify methylation markers, high‐depth WGBS was performed on surgically resected specimens, including 30 UC tumor tissues and 15 adjacent normal tissues, as well as urine samples obtained from 30 participants with non‐tumor urological diseases. The UC tissue set included 20 UCB and 10 UTUC samples (Figure 1A). Detailed clinicopathological characteristics of the tissue samples are presented in Table 1 and Table S1.

**TABLE 1:** Demographic and clinicopathologic characteristics of the participants.

Parameters and category		Discovery cohort			Validation cohort		Independent cohort	
		Non‐cancer	Adjacent normal	UC	Non‐cancer	UC	Non‐cancer	UC
Type and number of samples		urine (*n* = 30)	tissue (*n* = 15)	tissue (*n* = 30)	urine (*n* = 72)	urine (*n* = 93)	urine (*n* = 23)	urine (*n* = 32)
Age	Years, median (IQR)	67 (56–70)	64 (59–71)	62 (62–74)	62 (53–68)	66 (57–71)	66 (56–70)	66 (63–69)
Gender	Male, n (%)	25 (83.3)	11 (73.3)	21 (70.0)	56 (77.8)	73 (79.3)	20 (87.0)	21 (65.6)
Grade	HG, n (%)			25 (83.3)		73 (78.5)		23 (71.9)
T stage								
	pTa, n (%)			0 (0.0)		6 (6.4)		3 (9.4)
	pTis, n (%)			0 (0.0)		1 (1.1)		1 (3.1)
	pT1, n (%)			19 (63.3)		71 (76.3)		17 (53.1)
	pT2, n (%)			8 (26.7)		9 (9.7)		5 (15.6)
	pT3/4, n (%)			3 (10.0)		6 (6.5)		6 (18.8)
Disease type								
	UCB, n (%)		12 (80.0)	20 (66.7)		71 (76.3)		14 (43.8)
	UTUC, n (%)		3 (20.0)	10 (33.3)		22 (23.7)		18 (56.2)
	PUNLMP, n (%)	0 (0.0)			3 (4.2)		0 (0.0)	
	Urothelial dysplasia, n (%)	0 (0.0)			1 (1.4)		2 (8.7)	
	UPUMP, n (%)	0 (0.0)			5 (6.9)		3 (13.0)	
	Inverted papilloma, n (%)	0 (0.0)			5 (6.9)		0 (0.0)	
	BPH, n (%)	15 (50.0)			28 (38.9)		11 (47.8)	
	Urolithiasis, n (%)	7 (23.3)			25 (34.7)		4 (17.4)	
	Hydronephrosis, n (%)	3 (10.0)			1 (1.4)		2 (8.7)	
	Urethral stricture, n (%)	3 (10.0)			3 (4.2)		1 (4.4)	
	Cystitis glandularis, n (%)	2 (6.7)			1 (1.4)		0 (0.0)	
Participating centers								
	PKUFH, n (%)	30 (100.0)	15 (100.0)	30 (100.0)	72 (100.0)	93 (100.0)	5 (21.7)	5 (15.6)
	BJFH, n (%)	0 (0.0)	0 (0.0)	0 (0.0)	0 (0.0)	0 (0.0)	12 (52.2)	15 (46.9)
	BJCH, n (%)	0 (0.0)	0 (0.0)	0 (0.0)	0 (0.0)	0 (0.0)	6 (26.1)	12 (37.5)

Abbreviation: UC, urothelial carcinoma; UCB, urothelial carcinoma of the bladder; UTUC, upper tract urothelial carcinoma; IQR, interquartile range; HG, high grade; PUNLMP, papillary urothelial neoplasm of low malignant potential; UPUMP, urothelial proliferation of unknown malignant potential; BPH, benign prostate hyperplasia; PKUFH, Peking University First Hospital; BJFH, Beijing Friendship Hospital; BJCH, Beijing Chao‐Yang Hospital.

Two prospective cohorts were subsequently assembled for diagnostic evaluation between November 11, 2022, and May 14, 2025. The internal validation cohort comprised 165 participants (93 UC and 72 non‐cancer) recruited at PKUFH between November 11, 2022, and August 17, 2023. The external multicenter validation cohort included 55 participants (32 UC and 23 non‐cancer) enrolled from PKUFH, BJFH, and BJCH through May 14, 2025. Demographic and clinicopathologic characteristics of the validation cohorts are provided in Table 1 and Tables S2 and S3.

### 3.2 Methylation Marker Discovery and Feature Set Selection

To enhance robustness against inter‐individual variability, we leveraged genomic linkage blocks defined from cell type–resolved, biologically coherent CpG sites to identify UC‐specific DMRs [26]. In total, 101 307 hypomethylated and 1580 hypermethylated DMRs were identified (Figure 1B). To minimize potential noise arising from excessive features and to optimize the performance of the deep learning classifier, we systematically evaluated the impact of varying numbers of DMRs on diagnostic performance using the validation cohort (Figure 1B). For hypomethylated regions, DMRs were ranked according to the magnitude of differential methylation, and selecting increasing numbers of top‐ranked DMRs from 100 to 5000 progressively enhanced model performance, with AUC improving from 0.9757 to 0.9899 on the validation set; classifier performance stabilized at around the top 2000 DMRs and sensitivity peaked, representing an optimal balance between diagnostic performance and feature redundancy (Figure 2A and Table S4). In contrast, classifiers constructed using hypermethylated regions (top 100–2000) exhibited inferior discriminative performance, achieving a maximum AUC of only 0.9653 (Figure S2A and Table S4). Therefore, the final model was constructed using the top 2000 hypomethylated DMRs.

**FIGURE 2:**
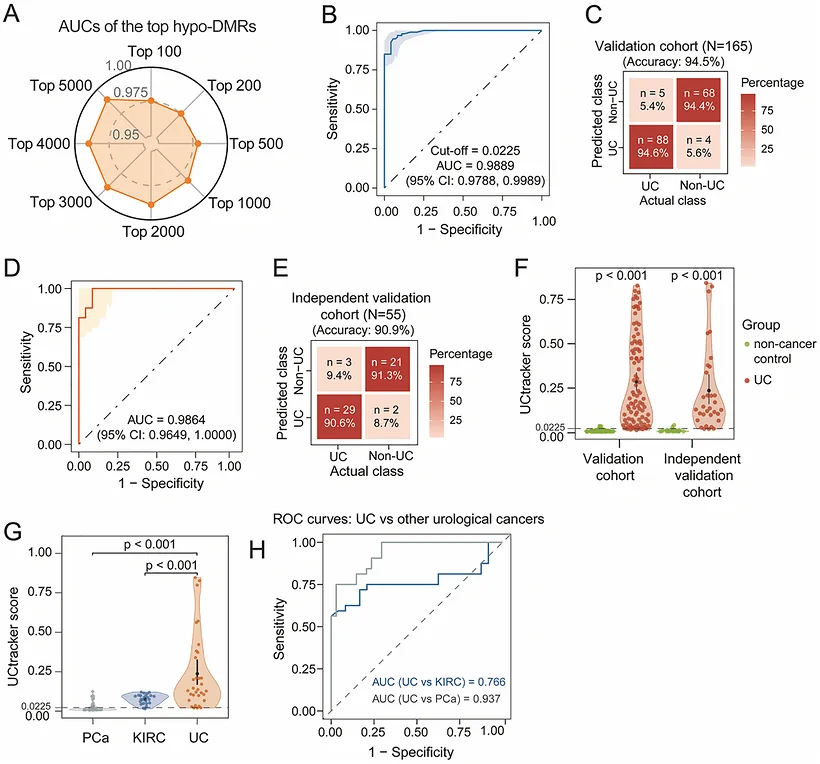
Diagnostic performance of the UCtracker model in validation cohorts. (A) Radar chart showing the number of selected hypomethylated (hypo) UC‐specific DMRs and the corresponding areas under the receiver operating characteristic curve (AUCs) in the validation cohort. (B) ROC curve of UC prediction in the validation cohort (*n* = 165). (C) Confusion matrices of UC prediction in the validation cohort. (D) ROC curve of UC prediction in the independent validation cohort (*n* = 55). (E) Confusion matrices of UC prediction in the independent validation cohort. (F) Comparison of UCtracker scores between UC patients and controls in the internal and independent validation cohorts. (G) Violin plots showing UCtracker scores for UC (*n* = 32), KIRC (*n* = 24), and PCa (*n* = 34) cohorts. (H) ROC curves for discriminating UC from KIRC and UC from PCa. KIRC, clear cell renal cell carcinoma; PCa, prostate cancer.

### 3.3 Tissue–Urine Concordance and Tumor Burden Validation of Model Score

Using the top 2000 hypomethylated DMRs, methylation levels showed a strong correlation between UC tissue and paired urine samples (R = 0.87, *p* < 2.2 × 10^−16^) (Figure S2B), supporting urine as a reliable surrogate for UC‐derived epigenetic alterations. Consistently, CNV profiles were highly concordant between UC tissue and paired urine samples (Figure S2C,D). These findings indicate that urine‐derived DNA robustly captures patient‐specific epigenetic and genomic characteristics of the primary tumor.

To further assess whether the model score reflects the abundance of UC‐derived signals, we performed an in silico read‐mixing analysis using high‐purity UC tumor tissue samples from the discovery cohort (Table S1) and non‐tumor urine samples from the independent cohort, as described in the Supplementary Methods. The model constructed by the top 2000 hypomethylated DMRs was applied to these mixtures without retraining. ROC analysis showed that discriminative performance significantly improved with increasing tumor content, with AUC values of 0.958 and 1.000 at tumor‐read proportions of 5% and ≥30%, respectively (Figure S2E). Consistently, model scores increased progressively with increasing tumor mixture ratio starting from 5% (Figure S2F). Together, these results support that model scores can serve as a proxy for malignancy burden in urine.

### 3.4 Functional Annotation of Hypomethylated DMRs

Genomic annotation of the top 2000 hypomethylated DMRs using ChIPseeker (v1.48.0) [32], as described in the Supplementary Methods, revealed that these regions were predominantly distributed in distal intergenic (37.8%) and intronic (32.6%) regions, with 6.15% mapping to promoter regions (Figure S3A and Table S5). Gene Ontology enrichment analysis of all annotated genes showed significant enrichment in biological processes related to developmental cell growth, cell–cell adhesion, and synaptic transmission (Figure S3B). KEGG pathway analysis further highlighted cadherin signaling and cAMP signaling (Figure S3C). Given their regulatory significance, promoter‐associated genes were enriched in pathways including cellular respiration, RNA polymerase complex, and ribosome biogenesis (Figure S3D,E). Notably, expression validation using GEPIA2 (http://gepia2.cancer‐pku.cn) confirmed that several promoter‐associated genes, e.g., *RAB3IP*, *NME1*, *GJB7*, *MKRN2OS*, *WDR72*, *CHRNA5*, and *MAPK15*, were significantly upregulated in tumor tissues compared to normal controls (Figure S3F–L). Together, these findings provide a biologically grounded interpretation of the model's underlying features, enhancing its plausibility for clinical applications. However, whether these methylation alterations are directly causal to tumorigenesis or represent downstream effects requires further experimental validation.

### 3.5 Validation of the UCtracker Model for UC Detection

The optimal score cutoff for tumor detection was determined as 0.0225 using the Youden index, and the final locked model was termed UCtracker (Figure 1B). In the internal validation cohort (*n* = 165; Table 1 and Table S2), UCtracker achieved an average AUC of 0.9889 (95% CI: 0.9788–0.9989), with a sensitivity of 94.6% and a specificity of 94.4% (Figure 2B,C). The model was further evaluated in an independent validation cohort comprising 32 UC patients and 23 benign controls (Table 1 and Table S3). UCtracker maintained high diagnostic performance in this cohort, achieving an AUC of 0.9864 (95% CI: 0.9649–1.0000), with a sensitivity of 90.6%, a specificity of 91.3%, and an overall accuracy of 90.9% (Figure 2D,E). UCtracker scores were significantly higher in UC patients than in non‐cancer controls in both the validation and independent cohorts (both *p* < 0.001; Figure 2F).

To further evaluate diagnostic specificity against other genitourinary malignancies, we applied the locked UCtracker model to additional urine samples from patients with kidney renal clear cell carcinoma (KIRC; *n* = 24) and prostate cancer (PCa; *n* = 34); detailed sample information is provided in Table S6. UCtracker scores were significantly higher in UC samples than in both KIRC and PCa samples (both *p* < 0.001; Figure 2G). ROC analysis showed strong discrimination between UC and PCa (AUC = 0.937) and moderate discrimination between UC and KIRC (AUC = 0.766) (Figure 2H). Collectively, these findings demonstrate that UCtracker is a reliable and accurate diagnostic tool for UC detection and shows potential for differentiating UC from other genitourinary malignancies.

### 3.6 Diagnostic Performance of UCtracker Across Clinical Subgroups and Compared With UroVysion FISH

UCtracker scores were significantly higher in patients with invasive tumors (≥ pT1) compared with those with non‐invasive disease (pTa/pTis) in the combined analysis (*p* < 0.001; Figure 3A), and this stage‐associated increase was consistently observed in both the validation cohort (*p* < 0.001; Figure S4A) and the independent cohort (*p* = 0.0016; Figure S4B). With respect to histological grade, UCtracker scores increased progressively with tumor aggressiveness, with significant differences between papillary urothelial neoplasm of low malignant potential (PUNLMP) and low‐grade (LG) tumors (*p* < 0.001), as well as between high‐grade (HG) and LG tumors (*p* = 0.001) (Figure 3B). These significant grade‐dependent differences were also observed in the cohort‐stratified analyses (Figure S4C,D). Consistently, tumors graded as G2 or G3 showed higher UCtracker scores than G1 tumors (*p* < 0.001; Figure S4E), whereas no significant difference was observed between UCB and UTUC (Figure S4F).

**FIGURE 3:**
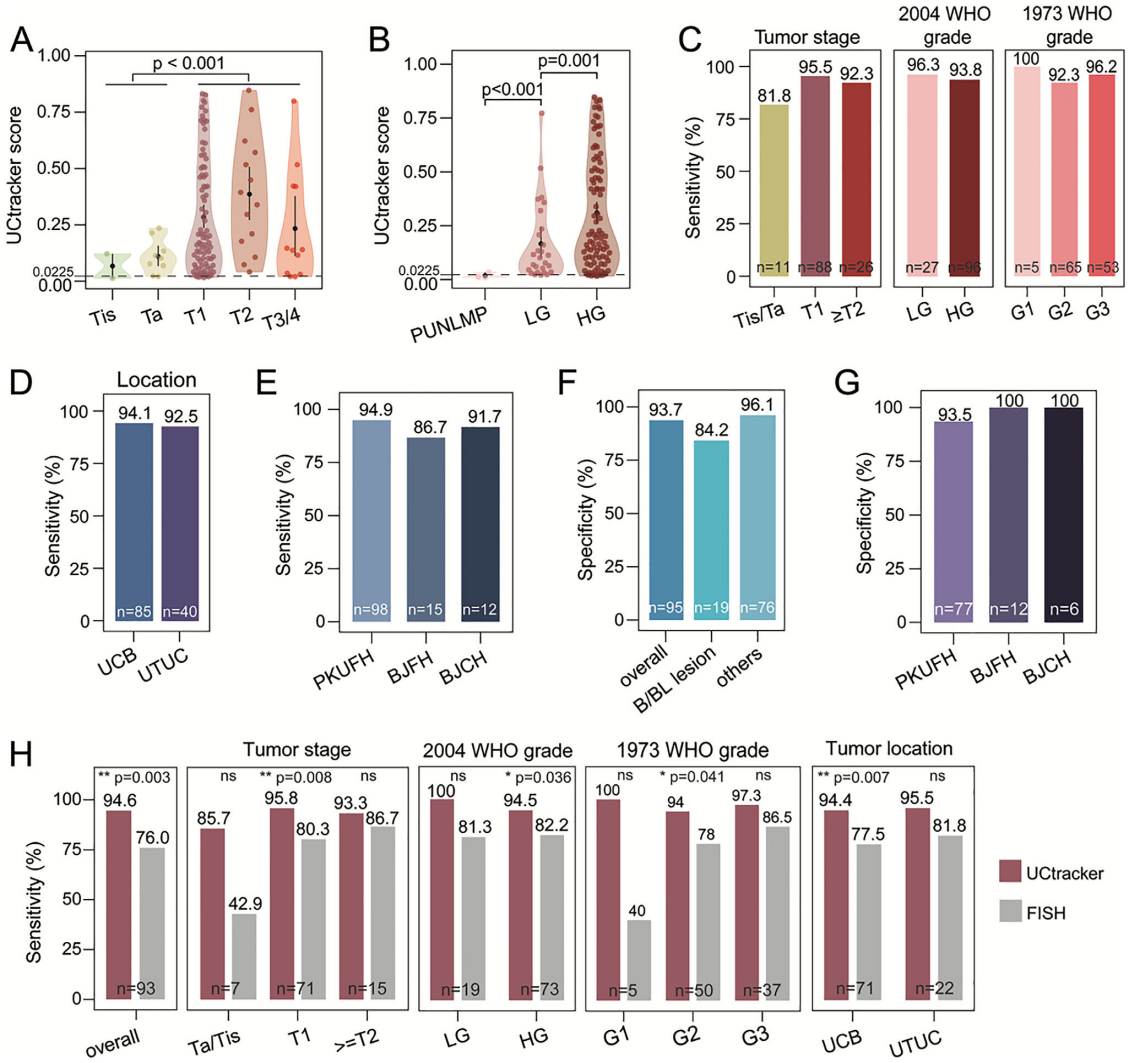
Performance of UCtracker across clinical subgroups and comparison with UroVysion FISH. (A, B) Distribution of UCtracker scores across pathological tumor stages (A) and tumor grade (B). (C) Sensitivity of UCtracker stratified by tumor T stage and grades. (D, E) Sensitivity of UCtracker stratified by tumor location (D) and participating center (E). (F) Specificity of UCtracker stratified by benign (B) and borderline (BL) tumor lesions and other non‐tumor conditions. (G) Specificity of UCtracker across participating centers. (H) Sensitivity comparison between UCtracker and FISH stratified by tumor stages, grades, and location. ns, not significant. PUNLMP, papillary urothelial neoplasm of low malignant potential; LG, low‐grade; HG, high‐grade; PKUFH, Peking University First Hospital; BJFH, Beijing Friendship Hospital; BJCH, Beijing Chao‐Yang Hospital.

UCtracker demonstrated robust diagnostic performance across pathological subgroups. In the combined analysis, sensitivity remained consistently high across tumor stages: 81.8% for non‐invasive disease (pTa/pTis), 95.5% for pT1, and 92.3% for muscle‐invasive tumors (≥pT2) (Figure 3C). Similar stage‐stratified sensitivities were observed in both the validation cohort and the independent cohort (Figure S4G,H). By histological grade, sensitivity was 96.3% for LG tumors and 93.8% for HG tumors, with further stratification showing sensitivities of 100%, 92.3%, and 96.2% for G1, G2, and G3 tumors, respectively (Figure 3C). Sensitivity was comparable between UCB and UTUC in the combined analysis (94.1% vs. 92.5%) and remained similar when the validation and independent cohorts were analyzed separately (Figure 3D and Figure S4G,H).

To assess potential center‐specific variability, we further evaluated UCtracker performance across participating centers. In the combined analysis, UCtracker maintained stable sensitivity of 94.9%, 86.7%, and 91.7% in PKUFH, BJFH, and BJCH, respectively (Figure 3E), and similar consistency was observed in cohort‐stratified analyses (Figure S4I). Regarding specificity, UCtracker effectively distinguished UC from benign or borderline tumor lesions (84.2%) and other non‐tumor conditions (96.1%) (Figure 3F). Notably, specificity remained consistently high and comparable across the three centers in both the integrated cohort and the independent cohort (Figure 3G and Figure S4J).

Matched UroVysion FISH data were available only in the validation cohort. For tumor stage, UCtracker demonstrated significantly higher sensitivity than FISH for pT1 tumors (95.8% vs. 80.3%, *p* = 0.003) (Figure 3H). Stratification by tumor grade revealed superior sensitivity of UCtracker for HG tumors (94.5% vs. 82.2%, *p* = 0.036), as well as for G2 tumors (94.0% vs. 78.0%, *p* = 0.041) (Figure 3H). Notably, UCtracker significantly outperformed FISH in UCB, achieving a sensitivity of 94.4% compared with 77.5% for FISH (*p* = 0.007) (Figure 3H). Although UCtracker showed numerically higher specificity than FISH in distinguishing UC from both benign and borderline tumor lesions and other non‐tumor conditions, these differences did not reach statistical significance (Figure S4K).

### 3.7 Robustness of UCtracker Performance Through Subsampling Analysis

To evaluate the robustness of UCtracker across varying sequencing depths, we performed systematic subsampling analyses in both the validation and independent validation cohorts. UCtracker scores derived from subsampled data showed strong concordance with scores obtained from original sequencing depth (3×–5×). At 1% and 10% subsampling, UCtracker scores were highly correlated with raw‐depth scores in the validation cohort (R = 0.9921 and R = 0.9993, respectively; both *p* < 2.2 × 10^−16^) and in the independent validation cohort (R = 0.9849 and R = 0.9994, respectively; both *p* < 2.2 × 10^−16^) (Figure 4A,B), indicating strong score stability under substantial depth reduction.

**FIGURE 4:**
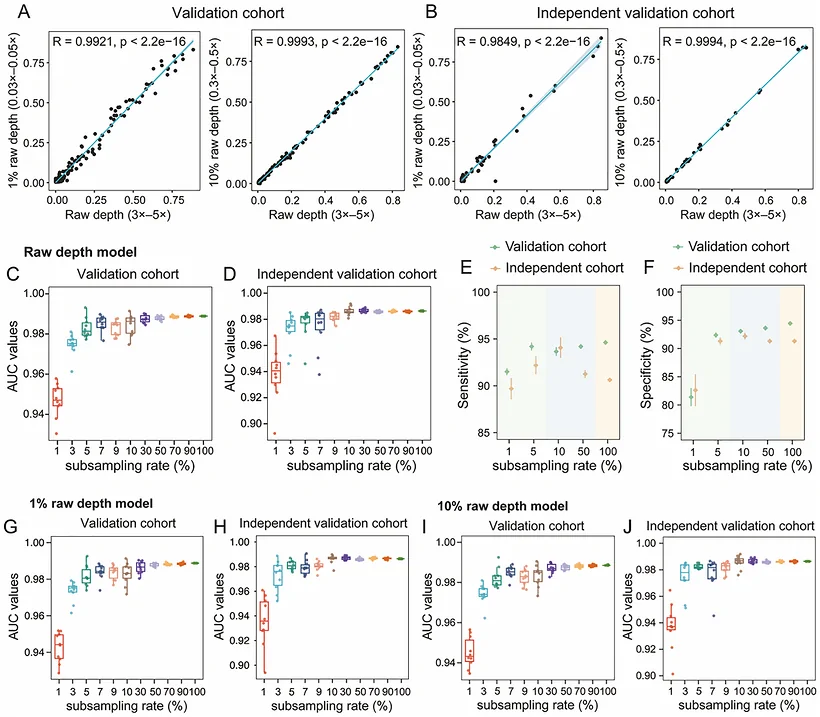
UCtracker exhibits high concordance and robust classification performance at low sequencing depths following subsampling analysis. (A, B) Pearson correlations between UCtracker scores derived from subsampled data and those obtained at raw sequencing depth (3×–5×) in the validation cohort (A) and independent cohort (B). (C, D) AUC values of the raw‐depth UCtracker model across a broad range of subsampling rates in the validation cohort (C) and independent cohort (D). (E, F) Diagnostic sensitivity (E) and specificity (F) across representative subsampling rates in the validation and independent cohorts. (G, H) AUC values of the 1% raw‐depth model evaluated across multiple subsampling rates in the validation cohort (G) and independent cohort (H). (I, J) AUC values of the 10% raw‐depth model evaluated across multiple subsampling rates in the validation cohort (I) and independent cohort (J).

We next evaluated cross‐depth diagnostic performance. First, the UCtracker model trained at the original sequencing depth was applied to datasets subsampled across a broad range of sequencing depths. At each subsampling depth, the UCtracker model was randomly trained and evaluated 10 times to ensure robustness. Discriminative performance was assessed by comparing AUC values across a broad range of subsampling rates. The AUC values showed only minor variation and remained consistently high, even at 10% of the original depth, in both the validation and independent validation cohorts (Figure 4C,D and Figure S5A,B). Diagnostic sensitivity, specificity, and accuracy were also stable across representative subsampling rates in both cohorts, with all three metrics consistently exceeding 90% at subsampling rates of 5% and above, and higher, and only a modest decline at the 1% subsampling level (Figure 4E,F and Figure S5C).

To further assess whether model performance depended on depth‐specific patterns, we trained additional models using 1% and 10% subsampled data and evaluated each fixed model across a consistent range of testing subsampling depths. These reduced‐depth models maintained stable AUC values across testing depths, particularly at subsampling rates of 10% and above, in both cohorts (Figure 4G–J and Figure S5D–G). Collectively, these results suggest that UCtracker primarily captures stable UC‐associated methylation signals rather than depth‐specific artifacts, supporting its applicability to urine WGBS data generated across variable sequencing depths.

### 3.8 Performance of UCtracker in Longitudinal Postoperative Surveillance of UC

To evaluate the performance of UCtracker in monitoring UC recurrence, we prospectively collected and analyzed serial postoperative urine samples (*n* = 131) from 48 eligible UC participants (Figure 1A,B). The median follow‐up duration is 216 days (interquartile range: 136–367). The UCtracker positive rate decreased markedly from 89.6% (43/48) preoperatively to 31.3% (15/48) postoperatively (Figure 5A). During postoperative longitudinally surveillance, 17 patients experienced intravesical recurrence confirmed by surgical pathology or biopsy, while 31 patients remained recurrence‐free throughout follow‐up (Figure 5A). Among patients with recurrence, 94.1% (16/17) were UCtracker‐positive. In the recurrence‐free cohort, UCtracker negativity was observed in 67.7% (21/31) of patients, with 61.3% (19/31) testing consistently negative throughout follow‐up (Figure 5A,B). In comparison, the UroVysion FISH assay detected 70.6% (12/17) of recurrent cases, while demonstrating a specificity of 95.5% (21/22) in recurrence‐free patients (Figure 5B). The clinical trajectory of each patient, annotated with pathological characteristics, longitudinal UCtracker and UroVysion FISH status, and clinical outcomes, is summarized in Figure 5C. Among patients who experienced recurrence, UCtracker positivity preceded clinical confirmation of recurrence by up to 250 days (Figure 5C), indicating its potential utility for early detection of residual or recurrent disease. The temporal concordance and divergence observed between UCtracker and UroVysion FISH further support the longitudinal stability and clinical reliability of UCtracker measurements.

**FIGURE 5:**
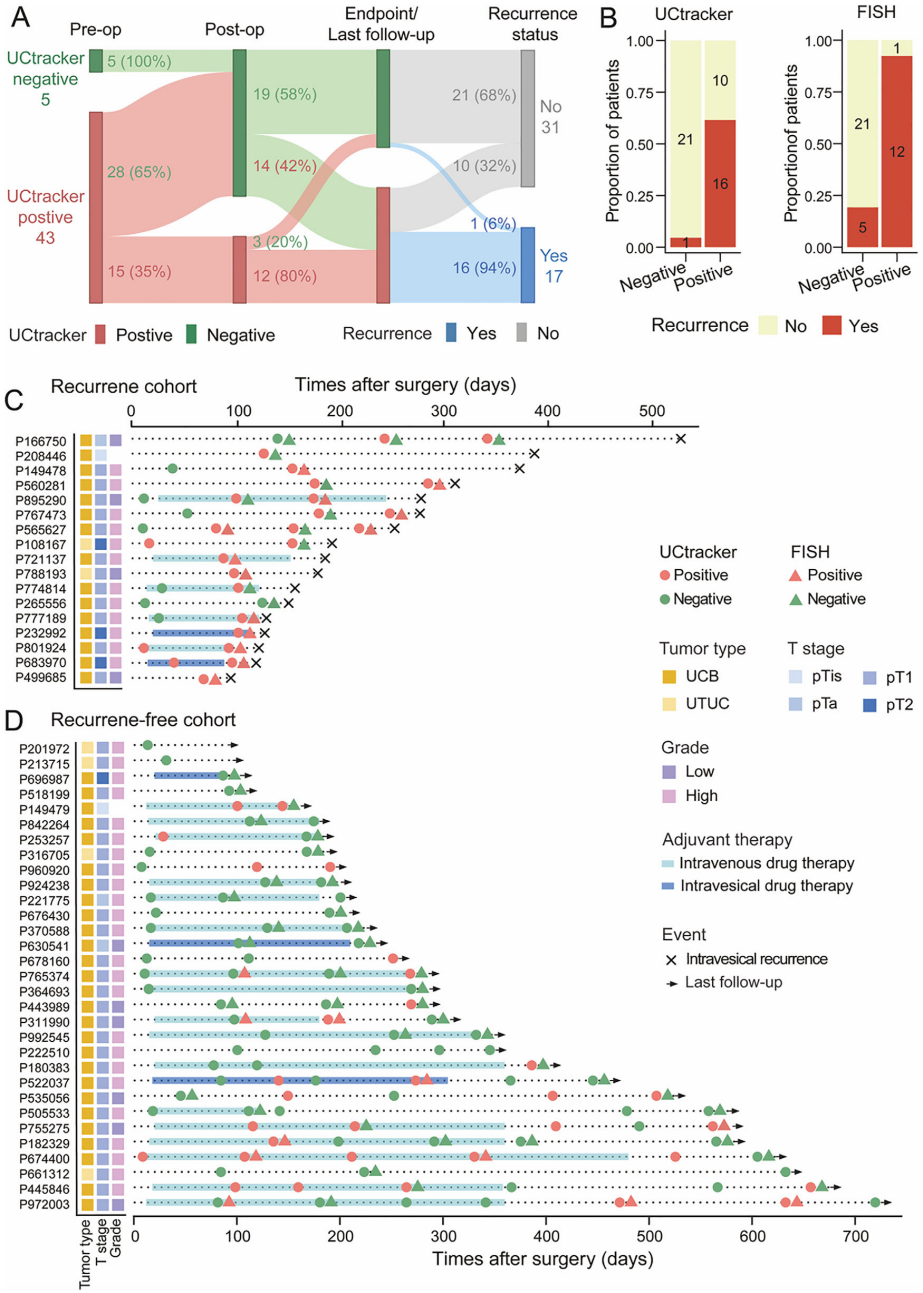
Longitudinal postoperative surveillance performance of UCtracker. (A) Sankey plot showing transitions in UCtracker status from the preoperative to postoperative period and their association with recurrence outcomes during follow‐up. Flow width is proportional to the number of patients in each category. (B) Proportions of UCtracker and UroVysion FISH positive and negative results stratified by recurrence status. (C, D) Swimmer plot showing longitudinal postoperative clinical trajectories of individual patients in the recurrence (C) and recurrence‐free cohorts (D), aligned by time after surgery. Longitudinal UCtracker and UroVysion FISH results are shown together with clinically relevant events, including adjuvant or intravesical therapy, intravesical recurrence, and last follow‐up. Heatmaps at the beginning of each bar indicate baseline pathological features.

### 3.9 UCtracker Score Dynamics Stratify Postoperative Recurrence Risk

Paired comparisons between preoperative and postoperative samples demonstrated a significant decline in UCtracker scores after surgery (Figure 6A). Patients who developed recurrence showed significantly higher UCtracker score log fold changes (postoperative vs. preoperative) than recurrence‐free patients (*p* = 0.019) (Figure 6B), suggesting that postoperative UCtracker dynamics are also associated with recurrence risk. To assess the robustness of these associations and reduce overfitting, we first performed univariable Cox regression for candidate variables, followed by 1000 bootstrap resampling iterations. UCtracker score log fold change and AJCC stage showed significant and relatively stable associations with RFS and were therefore included in the final multivariable Cox model. UCtracker score log fold change (HR = 2.59, 95% CI: 1.05–6.38; p = 0.038) and AJCC stage (HR = 4.77, 95% CI: 1.36–16.81; *p* = 0.015) remained independently associated with RFS in multivariable analysis (Figure 6C and Figure S6A). During postoperative adjuvant therapy, longitudinal monitoring further showed that UCtracker score dynamics tracked disease status, with persistently low scores in recurrence‐free patients (Figure 6D).

**FIGURE 6:**
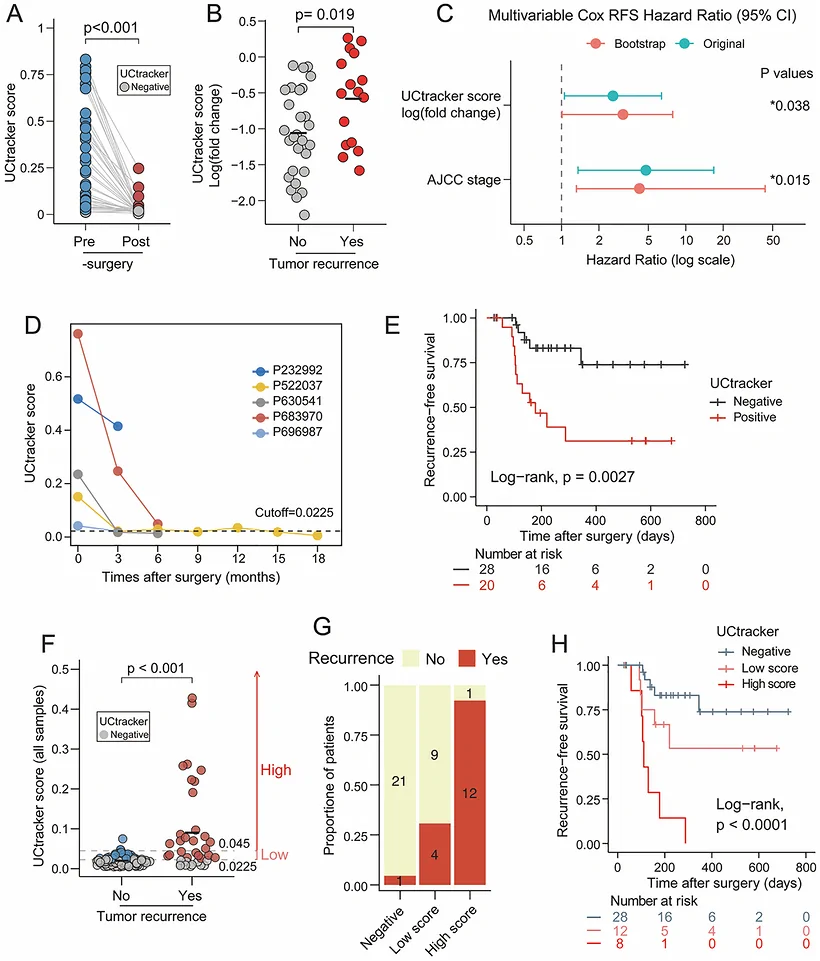
UCtracker score dynamics and risk stratification for postoperative recurrence. (A) Paired comparison of UCtracker scores between matched preoperative and postoperative samples using the Wilcoxon signed‐rank test. (B) Comparison of UCtracker score log fold change (postoperative/preoperative score), stratified by recurrence status during follow‐up. (C) Multivariable Cox regression analysis for recurrence‐free survival (RFS), showing hazard ratios and 95% confidence intervals for UCtracker score log fold change and AJCC stage, with original and bootstrap estimates. (D) Longitudinal UCtracker scores trajectories in five representative patients receiving postoperative adjuvant therapy. (E) Kaplan‐Meier curve of RFS stratified by postoperative landmark UCtracker status. (F) Distribution of UCtracker scores across all postoperative samples; the optimized cutoff of 0.045 was used to stratify UCtracker‐positive samples into low‐score and high‐score groups. (G) Proportion of recurrence and recurrence‐free patients in the UCtracker‐negative, low‐score, and high‐score groups. (H) Kaplan‐Meier analysis of RFS stratified by postoperative landmark UCtracker score classification.

We next evaluated whether stratifying postoperative UCtracker‐positive samples could improve recurrence risk discrimination. Prior to stratification, binary UCtracker positivity at postoperative landmark time points (1–3 months) was significantly associated with worse RFS, as demonstrated by Kaplan–Meier analysis (Figure 6E; log‐rank *p* = 0.0027) and univariable Cox regression (HR = 4.33, 95% CI: 1.52–12.32; *p* = 0.006). Using an optimized cutoff of 0.045, UCtracker‐positive samples were further stratified into low‐score and high‐score groups (Figure 6F and Figure S6B). The high‐score group achieved a substantially higher specificity for identifying recurrence (96.8% (30/31), Figure 6G), compared with the overall UCtracker‐positive group (67.7% (21/31), Figure 5B). This sub‐stratification significantly improved prognostic resolution, as shown by enhanced separation of RFS curves in Kaplan–Meier analysis (Figure 6H; *p* < 0.001). These results indicate that postoperative UCtracker score stratification provides a more refined assessment of recurrence risk than binary classification, supporting its potential clinical utility in postoperative surveillance and decision‐making.

### 3.10 Potential Recommendations for UCtracker‐Guided UC Recurrence Surveillance

Based on these findings, we propose a UCtracker‐guided strategy for risk‐adapted surveillance of UC recurrence (Figure 7). Integration of preoperative UCtracker status with postoperative landmark testing at 1–3 months enables dynamic recurrence risk stratification and may inform surveillance intensity. Patients who remain UCtracker‐negative at both time points are classified as very low risk and may be considered for de‐escalated follow‐up. Patients who convert to or remain UCtracker‐negative after surgery are considered low risk and may undergo less intensive cystoscopic and imaging surveillance, particularly if subsequent UCtracker results remain negative. In contrast, postoperative UCtracker positivity indicates increased recurrence risk. Patients with low positive scores are categorized as intermediate risk and may require closer surveillance, whereas those with high positive scores represent a high‐risk subgroup for whom prompt cystoscopy, intensified imaging, and consideration of early re‐intervention may be warranted.

**FIGURE 7:**
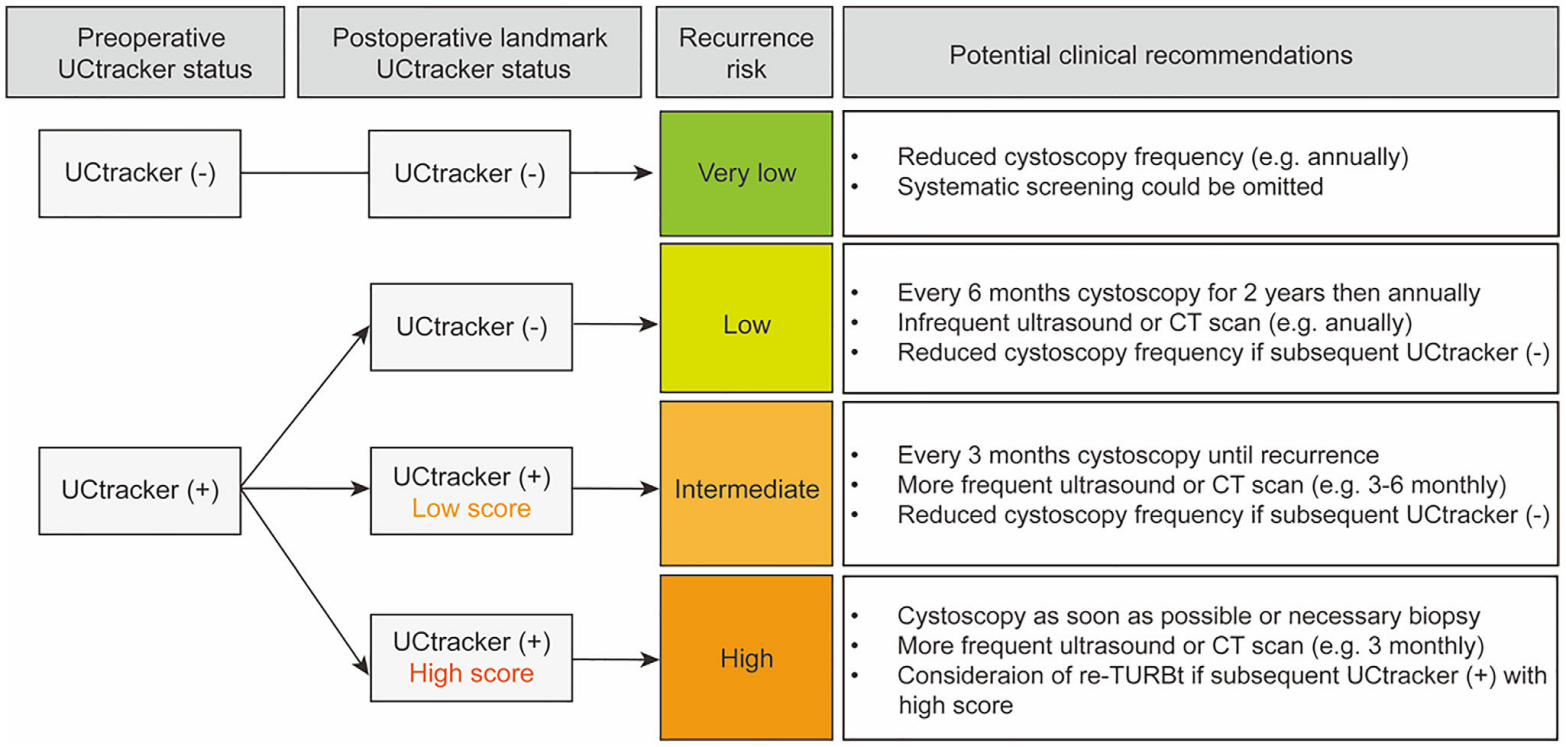
Proposed schema for UCtracker‐guided recurrence surveillance strategy in UC. Preoperative and postoperative UCtracker status, together with postoperative score classification, are integrated to stratify recurrence risk and guide potential risk‐adapted surveillance strategies. CT, computed tomography; re‐TURBt, repeated transurethral resection of bladder tumor.

## 4 Discussion

Given that UCtracker was developed from pooled UCB and UTUC samples, demonstrating their epigenetic similarity is essential to validate its pan‐urothelial applicability. While early gene‐specific studies suggested more extensive promoter hypermethylation in UTUC [14, 33, 34], recent epigenome‐wide profiling has consistently revealed highly concordant DNA methylation patterns between the two sites. Our previous WGBS of tumor specimens and urinary sediment demonstrated that UTUC and UCB share virtually identical methylation profiles, dominated by a common repertoire of cancer‐specific epigenetic alterations [12]. An independent comparative methylome analysis corroborated these aligned DNA methylation landscapes [25]. Such epigenetic concordance provides a robust biological rationale for the joint modeling of UCB and UTUC and strongly supports the pan‐urothelial applicability of UCtracker.

UCtracker introduces several key methodological advances over our previous UCseek framework [13], including the transition from urine sediment to whole urine for more comprehensive tumor DNA capture, which integrates both cfDNA and utDNA; the adoption of single‐stranded post‐bisulfite conversion library preparation to eliminate position‐dependent methylation bias; and the implementation of a CNN–BiLSTM deep learning architecture for more accurate read‐level classification. Previous studies have demonstrated that both tissue‐derived and urinary cfDNA methylation profiles sensitively reflect tumor activity, cellular origin, and minimal residual disease [35, 36, 37]. Moreover, urinary cfDNA exhibits high concordance in somatic mutations and copy number alterations, while each fraction may capture complementary biological aspects of tumor burden and disease dynamics [38]. Consistent with this rationale, we observed strong concordance between DNA methylation and CNV profiles of UC tissues and paired urine samples in our study (Figure S2C,D), further supporting the integration of these two DNA compartments as a more reliable surrogate for the tumor epigenetic landscape.

UC‐specific DMRs in this study were identified through systematic comparisons between UC tissue samples and controls, including matched adjacent normal tissues and urine samples from patients with benign urological conditions. By subtracting methylation signals present in benign control urine, we effectively minimized urine background noise arising from normal urothelial turnover and other non‐tumor sources, thereby enriching for UC‐specific methylation alterations and improving the robustness of marker selection. The robust performance across cohorts of UCtracker can be also attributed to the use of biologically defined methylation linkage blocks derived from a comprehensive normal cell‐type atlas, which improves signal‐to‐noise discrimination and reduces susceptibility to stochastic methylation variation [26].

We observed that hypomethylated DMRs substantially outperformed hypermethylated regions for UC detection (Figure 2A and Figure S2A), consistent with prior studies showing that global hypomethylation is a hallmark of UC and contributes to genomic instability and malignant progression [39, 40, 41, 42]. Wolff et al. [43] found that noninvasive UC exhibits a distinct pattern of hypomethylation, whereas invasive tumors are characterized by widespread hypermethylation. Notably, they also identified hypermethylation at 12% of loci in normal‐appearing urothelium from cancer‐bearing bladders, supporting the presence of an epigenetic field defect. Accordingly, hypomethylated markers may capture early tumor‐associated events while being less affected by background hypermethylation in normal‐appearing urothelium. Collectively, these findings support that UCtracker, by leveraging hypomethylated DMRs, preferentially detects early tumorigenic epigenetic alterations, thereby enabling sensitive UC detection. Future subtype‐exclusion training experiments, in which the model is trained on all but one subtype and tested on the excluded subtype, could further determine whether model performance reflects subtype‐specific methylation patterns or more general early hypomethylation signals.

Compared to the UroVysion FISH test, UCtracker showed significantly superior sensitivity in several clinically relevant subgroups, including pT1 tumors, high‐grade tumors, and UCB (Figure 3H). This advantage likely reflects the fundamental biological properties of DNA methylation alterations, which represent early, stable, and cumulative events during carcinogenesis [44, 45], and therefore provide a broader and more consistent detection window than cytology‐based assessment or chromosomal instability detected by UroVysion FISH. Notably, UCtracker maintained a sensitivity exceeding 80% even in non‐invasive disease (pTa/pTis) (Figure 3C), highlighting its potential utility for early tumor detection and justifying further evaluation in prospective screening studies among high‐risk populations.

Another advantage of UCtracker lies in the integration of genome‐wide bisulfite sequencing with a deep learning framework (CNN‐BiLSTM) that explicitly models read‐level methylation patterns within UC‐specific hypomethylated regions [16, 27]. In contrast to single‐gene or small‐panel methylation assays, which can be limited by tumor heterogeneity, UCtracker captures a more comprehensive epigenetic signature of UC. Importantly, UCtracker exhibited remarkable robustness to sequencing depth reduction, even when only 1% of the original reads were retained. UCtracker scores remained highly correlated with those derived from full‐depth data, and diagnostic performance was largely preserved (Figure 4). This stability at ultralow sequencing depth substantially lowers the economic and technical barriers to implementation, enabling cost‐effective early detection and supporting the applicability of UCtracker across diverse clinical settings.

For recurrence surveillance, UCtracker detected 94.1% of recurrences and provided a lead time of up to 250 days prior to clinical confirmation (Figure 5C). This early‐window capability could create opportunities for timely therapeutic intervention. Beyond binary classification, the analysis of postoperative UCtracker score dynamics and the establishment of a refined stratification cutoff (0.045) enhanced risk discrimination (Figure 6F–H). This allows for differentiation between patients with residual molecular disease at high risk of clinical recurrence and those with low‐level background signals, potentially reducing unnecessary invasive procedures (e.g., cystoscopy) in the latter group while intensifying surveillance for the former.

Several limitations should be acknowledged. First, although a multicenter prospective design was employed, the overall sample size was modest and predominantly contributed by a single center. Therefore, larger prospective multicenter studies with more balanced enrollment across centers are needed to further validate the generalizability of UCtracker for UC detection and recurrence monitoring. Second, although UCtracker was further evaluated against KIRC and PCa cohorts, the number of non‐UC genitourinary malignancy samples remains limited, and differences in library preparation may have influenced cross‐cancer comparisons. Larger cohorts of other urological malignancies processed using harmonized experimental protocols are required to further validate the differential diagnostic specificity of UCtracker. Third, the postoperative surveillance cohort was small with few recurrence events and short follow‐up, raising concerns about overfitting. Although bootstrap resampling supported robustness, these findings should be interpreted cautiously and validated in larger, multicenter cohorts. Fourth, the comparative analyses with UroVysion FISH were performed using the same validation datasets rather than a fully independent external test set; this may introduce optimistic bias in the estimated sensitivity and lead‐time benefits and warrants cautious interpretation. Finally, despite the demonstrated analytical robustness at ultralow sequencing depths, a formal cost‐effectiveness analysis in real‐world clinical settings is still required to inform optimal implementation strategies.

In conclusion, UCtracker is an ultra‐accurate and robust deep learning‐based DNA methylation model for the diagnosis and postoperative monitoring of UC. Its consistent performance at low sequencing depths, coupled with its capacity to provide dynamic, risk‐stratified molecular residual disease insights, underscores its potential for cost‐effective and personalized management throughout the UC care continuum.

## Author Contributors

S.X., G.L., Y.W., Y.L., and H.G. contributed equally to this work as co‐first authors. L.Z., W.C, X.L. and S.H. conceived and designed the study. S.X, G.L., Y.W., H.G., Y.Z., G.X., Y.L., B.G., Y.S., L.T., X.Z., Y.T., Z.J., M.X., J.F., Z.L. and Y.G. contributed to data collection, curation, and interpretation. S.W., G.L., and Y.L. analyzed the acquired data. S.W., and G.L. drafted the original manuscript. L.Z., W.C., X.L., S.H. and Y.G. critically reviewed and revised the manuscript for important intellectual content. All authors read and approved the final manuscript.

## Conflicts of Interest

The authors declare no conflicts of interest.

## Supporting information




**Supporting File 1**: advs76710‐sup‐0001‐SuppMat.docx.

**Supporting File 2**: advs76710‐sup‐0002‐TableS1‐S6.xlsx.

## Data Availability

The tissue and urinary original sequencing data in this study have been deposited in the Genome Sequence Archive (GSA) for human under the accession number HRA016034 and PRJCA055535 at https://ngdc.cncb.ac.cn/gsa/. Access can be requested from the corresponding author upon reasonable request.
