# Supplementary material for: UCtracker: A Deep Learning–Based DNA Methylation Model for Noninvasive Diagnosis and Recurrence Surveillance of Urothelial Carcinoma in a Prospective Study
Source: Adv Sci (Weinh). 2026 Aug 3:e76710. Online ahead of print. doi: 10.1002/advs.76710 (PMC13430930; doi:10.1002/advs.76710)
Supplement: Supplementary file 1 — Supporting File 1: advs76710‐sup‐0001‐SuppMat.docx. [file ADVS-9999-e76710-s001.docx]

**S****upplementary materials**

**UCtracker: A Deep Learning–Based DNA Methylation Model for Noninvasive Diagnosis and Recurrence Surveillance of Urothelial Carcinoma in a Prospective Study**

**TABLE OF CONTENTS**

**Supplementary Methods**

- Participants enrollment
- Sample size determination
- Copy-number variation analysis
- Genomic annotation of DMRs
- GO and KEGG enrichment analysis
- In silico read-mixing analysis for evaluating the effect of tumor content
- Bootstrap internal validation for Cox regression models

**Supplementary Figures**

- Figure S1 Schematic overview of the UCtracker deep learning framework
- Figure S2 Tissue–urine molecular concordance and tumor-content-dependent validation of UCtracker scores
- Figure S3 Genomic annotation and functional enrichment of top 2,000 hypomethylated DMRs
- Figure S4 Cohort-stratified clinical subgroup performance of UCtracker and comparison with UroVysion FISH
- Figure S5 Stability of UCtracker diagnostic performance across subsampling depths
- Figure S6 Prognostic value of UCtracker scores and stratification of postoperative UCtracker scores

**Supplementary Tables**

- Table S1 Clinical characteristics and WGBS sequencing information of the discovery cohort
- Table S2 Clinical characteristics of the validation cohort
- Table S3 Clinical characteristics of the independent validation cohort
- Table S4 Diagnostic performance of different top-ranked hypermethylated (hyper) and hypomethylated (hypo) DMR sets
- Table S5 Genomic annotation of the top 2,000 hypomethylated DMRs selected for UCtracker model training
- Table S6 Clinical characteristics of the KIRC and PCa cohort

**Supplementary Methods**

**Participants enrollment**

Participants in the study were prospectively recruited from three centers: Peking University First Hospital (PKUFH), Beijing Friendship Hospital (BJFH) and Beijing Chao-Yang Hospital (BJCH). Participating centers were qualified to conduct clinical research in accordance with Good Clinical Practice (GCP) guidelines. The enrollment period spanned from November 11, 2022, to May 14, 2025. The study was conducted in accordance with the principles of the Declaration of Helsinki, and written informed consent was obtained from all participants prior to enrollment.

Participants with a clinical suspicion of urothelial carcinoma (UC) were prospectively enrolled based on the following inclusion criteria: (1) age ≥18 years; (2) radiological or pathological findings suggestive of primary UC, with planned surgical treatment; and (3) provision of written informed consent and availability of tissue or urine samples for the study. Participants were excluded if they had contraindications to surgery, such as pregnancy, lactation, or hemorrhagic disorders, which precluded definitive pathological confirmation. Participants were also excluded if urine samples yielded insufficient DNA after extraction or failed sequencing quality control. In addition, patients scheduled for radical cystectomy (RC) in the short term or those lost to follow-up, rendering completion of recurrence surveillance infeasible, were excluded.

Relevant clinical information, including demographic data, diagnosis findings (CT, MRI, ultrasonography, cystoscopy, etc., when available), and clinicopathological characteristics, was systematically collected for each participant. Tumor stage and grade were determined according to the 8th edition of the American Joint Committee on Cancer (AJCC) cancer staging and the 2004/2016 World Health Organization (WHO) grading system, respectively [1, 2].

**Sample size determination**

Sample size estimation was performed in accordance with the STARD guidelines for diagnostic accuracy studies and was based on demonstrating the superiority of UCtracker over UroVysion fluorescence in situ hybridization (FISH) in terms of diagnostic sensitivity for UC. The primary accuracy parameter was sensitivity. The null and alternative hypotheses were defined as follows:

Null hypothesis (H₀): The sensitivity of UCtracker is equal to or lower than that of FISH.

Alternative hypothesis (H₁): The sensitivity of UCtracker is higher than that of FISH.

Based on preliminary data, the anticipated sensitivity of UCtracker (P_T_) was 0.93 [3], while the reference sensitivity of FISH (P_0_) was 0.75 [4]. The statistical power was set at 90% (1 − β = 0.90), with a two-sided significance level of 0.05. The required number of UC-positive participants was calculated using a normal approximation for comparing an expected sensitivity against a reference value:

$$n=\frac{\left( Z_{1-\alpha/2} + Z_{1-\beta} \right)^{2}\times P_{T}(1-P_{T})}{\left( P_{T} - P_{0})^{2} \right.}$$

where n denotes the number of participants with pathologically confirmed UC, and $Z_{1-\alpha/2}$and $Z_{1-\beta}$ represent the standard normal quantiles corresponding to the predefined significance level and power, respectively.

This calculation indicated that 82 UC-positive cases were required. Assuming a 1:1 ratio of UC-positive to UC-negative participants, a total of 164 evaluable participants were needed. To account for potential exclusions due to inadequate sample quality, sequencing failure, or loss to follow-up, the target sample size was increased to 180 participants, comprising 90 UC-positive cases and 90 UC-negative controls.

**Copy-number variation analysis**

Genome-wide read coverage was summarized into non-overlapping 1-Mb bins using readCounter from the HMMcopy suite [5]. Bin-level coverage profiles were processed with ichorCNA [6], with GC-content and mappability correction based on precomputed hg19 reference tracks, to infer copy-number states, ploidy, and tumor fraction. The resulting copy-number profiles were used for visualization of CNV landscapes and quantitative assessment of tumor fraction.

**Genomic annotation of DMRs**

Genomic annotation of the top 2,000 hypomethylated DMRs was performed using the R package ChIPseeker (version 1.48.0) [7]. The DMR coordinates first converted to a standard BED file format containing chromosome, start, and end positions. Annotation was carried out against the human reference genome hg19, using the *TxDb.Hsapiens.UCSC.hg19.knownGene* transcript database and the *org.Hs.eg.db* gene annotation package. Promoter regions were defined as ±3 kb from the transcription start site (TSS). The *annotatePeak* function was used to assign each DMR to genomic features, including promoter, exon, intron, downstream, and distal intergenic regions, according to its position relative to annotated genes. The resulting annotation table, including genomic location, nearest gene, distance to TSS, and functional genomic category, was exported for downstream summary and visualization.

**GO and KEGG enrichment analysis**

Genes associated with the annotated DMRs were extracted from the ChIPseeker annotation results using Entrez gene IDs. After removing missing values and duplicate entries, Gene Ontology (GO) enrichment analysis was performed using the *enrichGO* function from the *clusterProfiler* R package (version 4.14.6). The analysis was conducted separately for three GO ontologies: Biological Process (BP), Molecular Function (MF), and Cellular Component (CC). Statistical significance was defined with a Benjamini‑Hochber adjusted p‑value cutoff of 0.05 and a q‑value cutoff of 0.05. The same list of Entrez IDs was subjected to Kyoto Encyclopedia of Genes and Genomes (KEGG) pathway enrichment using *enrichKEGG* with the human organism annotation (*hsa*) and the same multiple-testing correction strategy. Finally, the top enriched terms or pathways were ranked by adjusted p‑value for downstream visualization.

**In silico read-mixing analysis for evaluating the effect of tumor content**

To investigate whether locked model scores reflect the fraction of tumor-derived signals in urine samples, we performed an in silico read-mixing analysis using tumor tissue samples from the discovery cohort and non-tumor urine samples from the independent cohort. Tumor purity for each UC tissue sample was first estimated by CNV analysis using ichorCNA. Six UC tissue samples with high estimated tumor purity, defined as tumor fraction greater than 85%, were selected as tumor-derived sources for the mixing analysis (Table S1).

For each simulation, sequencing reads from a high-purity tumor tissue sample and a non-tumor urine sample were randomly sampled and mixed at predefined tumor-read proportions, including 0.1%, 0.5%, 1%, 5%, 10%, 30%, 60%, and 90%. The total number of reads in each simulated sample was fixed at 10 million reads to exclude the confounding effect of sequencing depth. For each mixing proportion, the corresponding number of reads was sampled from the tumor tissue sample, and the remaining reads were sampled from the non-tumor urine background. The mixed reads were then processed using the same data preprocessing and feature extraction pipeline.

The model constructed by the top 2,000 hypomethylated DMRs, with no further retraining or parameter adjustment, was applied to each simulated mixture to generate model scores and the estimated fraction of UC-derived reads. The relationship between the predefined tumor-read proportion and the model score was assessed to determine whether the model output increased in a tumor-content-dependent manner. This analysis was designed to evaluate whether the scores reflect changes in tumor-derived signals abundance and to support the interpretation of the model score as a proxy for malignancy burden.

**Bootstrap internal validation for Cox regression models**

To assess the robustness of the univariable and multivariable Cox proportional hazards regression analyses, we performed an internal validation using Bootstrap resampling with 1,000 iterations. For each iteration, a Bootstrap sample of the same size as the original recurrence surveillance cohort (n = 48) was drawn with replacement from the original data. Univariable and multivariable Cox regression models were refitted on each Bootstrap sample, and hazard ratios (HRs) with 95% confidence intervals (CIs) were calculated. The distribution of HRs and CIs across the 1,000 Bootstrap iterations was used to evaluate the stability of the original estimates. Variables with statistically significant associations (*P* < 0.05) and stable CIs in the univariable analysis were selected for inclusion in the multivariable model. Bootstrap validation was performed using the R package *boot* (version 1.3-32).





**Figure S1** **Schematic overview of the UCtracker deep learning framework.** (A) Workflow for UC-specific DMR extraction. WGBS data from UC tumor tissues, UC-adjacent normal tissues, and non-cancer urine samples were processed using paired-end sequencing and adapter trimming, followed by methylation atlas-guided DMR block screening and differential methylation analysis to identify UC-specific DMRs. (B) Read-level methylation encoding. Each 140-bp read overlapping the selected UC-specific DMRs was represented as a 140 × 5 matrix encoding nucleotide identity and methylated cytosine status. (C) CNN–BiLSTM read-level model. The encoded read matrix was processed through convolutional layers, max-pooling, dropout, bidirectional LSTM, and fully connected layers to generate a read-level p-score, representing the probability that the read was UC-derived. (D) MLE integration. Read-level p-scores from all informative reads within each sample were integrated using maximum likelihood estimation to derive a sample-level tumor fraction score. (E) Optimal cutoff determination. The decision threshold was determined by ROC analysis by maximizing Youden’s J statistic, thereby selecting the cutoff with the optimal combined sensitivity and specificity for tumor detection.





**Figure S2** **Tissue–urine molecular concordance and tumor-content-dependent validation of UCtracker scores**. (A) Diagnostic performance of classifiers constructed using different numbers of top-ranked hypermethylated UC-specific DMRs in the validation cohort. (B) Correlation of methylation levels between UC tissue and paired urine samples based on the top 2,000 hypomethylated DMRs. (C, D) Representative genome-wide CNV profiles of tumor tissue (C) and matched urine sample (D). (E) ROC curves of the locked model in simulated mixtures of tumor-derived and non-tumor urine reads at predefined tumor-read proportions. (F) Distribution of model p-scores across tumor mixture ratios.





**Figure S3 Genomic annotation and functional enrichment of top 2,000 hypomethylated DMRs**. (A) Genomic distribution of the top 2,000 hypo-DMRs. (B, C) GO (B) and KEGG (C) enrichment analyses of all annotated genes. (D, E) GO (D) and KEGG (E) enrichment of promoter-associated genes. (F–L) Expression levels of selected promoter-associated gene in tumor versus normal tissues based on GEPIA2 (BLCA). **P* < 0.01; T, tumor; N, normal.





**Figure S4** **Cohort-stratified clinical subgroup performance of UCtracker and comparison with UroVysion FISH.** (A, B) Distribution of UCtracker scores across tumor stages in the validation cohort and independent cohort, respectively. (C, D) Distribution of UCtracker scores across 2004 WHO grade groups in the validation cohort and independent cohort, respectively. (E) Distribution of UCtracker scores across 1973 WHO grade groups. (F) Distribution of UCtracker scores between UCB and UTUC. (G, H) Sensitivity of UCtracker stratified by tumor T stage, 1973 WHO grade, 2004 WHO grade, and tumor location in the validation cohort (G) and in the independent cohort (H). (I, J) Sensitivity (I) and specificity (J) of UCtracker stratified by participating center in the independent cohort. (K) Specificity comparison between UCtracker and UroVysion FISH in the validation cohort, stratified by all non-cancer samples, benign (B) and borderline (BL) tumor lesions, and other non-tumor conditions. ns, not significant.





**Figure S5 Stability of UCtracker diagnostic performance across subsampling depths, related to Figure 4.** (A, B) Representative ROC curves of raw-depth model across multiple subsampling rates in the validation cohort (A) and independent cohort (B). (C) Diagnostic accuracy across representative subsampling rates in both cohorts. (D, E) Representative ROC curves of 1% raw-depth model across subsampling rates in the validation cohort (D) and independent cohort (E). (F, G) Representative ROC curves of 10% raw-depth model across subsampling rates in the validation cohort (F) and independent cohort (G).





**Figure S6** **Prognostic value of** **UCtracker scores and stratification of postoperative UCtracker scores, related to Figure 6.** (A) Univariable Cox regression analysis of recurrence-free survival (RFS) for UCtracker score log fold change, preoperative and postoperative UCtracker scores, tumor stage, tumor grade, age, and gender, with original and bootstrap estimates. (B) Distribution of UCtracker scores in postoperative samples collected at last follow-up or recurrence. The dashed line indicates the optimized cutoff of 0.045 used to stratify UCtracker-positive samples into low-score and high-score subgroups.

**Reference:**

1. Paner GP, Stadler WM, Hansel DE, Montironi R, Lin DW, Amin MB. Updates in the Eighth Edition of the Tumor-Node-Metastasis Staging Classification for Urologic Cancers. Eur Urol. 2018; 73: 560-9.

2. Netto GJ, Amin MB, Berney DM, Compérat EM, Gill AJ, Hartmann A, et al. The 2022 World Health Organization Classification of Tumors of the Urinary System and Male Genital Organs-Part B: Prostate and Urinary Tract Tumors. Eur Urol. 2022; 82: 469-82.

3. Wang P, Shi Y, Zhang J, Shou J, Zhang M, Zou D, et al. UCseek: ultrasensitive early detection and recurrence monitoring of urothelial carcinoma by shallow-depth genome-wide bisulfite sequencing of urinary sediment DNA. EBioMedicine. 2023; 89: 104437.

4. Chou R, Gore JL, Buckley D, Fu R, Gustafson K, Griffin JC, et al. Urinary Biomarkers for Diagnosis of Bladder Cancer: A Systematic Review and Meta-analysis. Ann Intern Med. 2015; 163: 922-31.

5. Shah SP, Xuan X, DeLeeuw RJ, Khojasteh M, Lam WL, Ng R, et al. Integrating copy number polymorphisms into array CGH analysis using a robust HMM. Bioinformatics. 2006; 22: e431-9.

6. Adalsteinsson VA, Ha G, Freeman SS, Choudhury AD, Stover DG, Parsons HA, et al. Scalable whole-exome sequencing of cell-free DNA reveals high concordance with metastatic tumors. Nat Commun. 2017; 8: 1324.

7. Yu G, Wang LG, He QY. ChIPseeker: an R/Bioconductor package for ChIP peak annotation, comparison and visualization. Bioinformatics. 2015; 31: 2382-3.
